# Three-dimensional conformal radiotherapy with concurrent chemotherapy for postoperative recurrence of esophageal squamous cell carcinoma: clinical efficacy and failure pattern

**DOI:** 10.1186/1748-717X-8-241

**Published:** 2013-10-18

**Authors:** Yong Bao, ShiLiang Liu, QiChao Zhou, PeiQiang Cai, Simone Anfossi, QiaoQiao Li, YongHong Hu, MengZhong Liu, JianHua Fu, TieHua Rong, Qun Li, Hui Liu

**Affiliations:** 1 State Key Laboratory of Oncology in South China, Sun Yat-sen University Cancer Center, Guangzhou, Guangdong, P.R. China; 2 Department of Radiation Oncology, Sun Yat-sen University Cancer Center, 651 Dongfengdong Road, Guangzhou, Guangdong 510060, P.R. China; 3 Department of Radiology, Sun Yat-sen University Cancer Center, Guangzhou, Guangdong, P.R. China; 4 Department of Thoracic Surgery, Sun Yat-sen University Cancer Center, Guangzhou, Guangdong, P.R. China; 5 Guangdong Esophogeal Cancer Research Institute, Guangzhou, Guangdong, P.R.China; 6 Department of Hematopathology, The University of Texas, MD Anderson Cancer Center, Houston, Texas, USA

**Keywords:** Esophageal squamous cell carcinoma, Postoperative recurrence, Concurrent chemoradiotherapy

## Abstract

**Background:**

To assess the therapeutic outcome and failure pattern of three-dimensional conformal radiotherapy (3D-CRT)-based concurrent chemoradiotherapy (CCRT) for recurrence of esophageal squamous cell carcinoma (SCC) after radical surgery.

**Methods:**

Treatment outcome and failure pattern were retrospectively evaluated in 83 patients with localized cervical and thoracic recurrences after radical surgery for thoracic esophageal SCC. All patients were treated with 3DCRT-based CCRT (median radiation dose 60 Gy), in which 39 received concurrent cisplatin plus 5-fluorouracil (PF), and 44 received concurrent docetaxel plus cisplatin (TP). Treatment response was evaluated at 1–3 months after CCRT.

**Results:**

With a median follow-up of 34 months (range, 2–116 months), the 3-year overall survival (OS) of all the patients was 51.8% and the median OS time was 43.0 months. The overall tumor response rate was 75.9% (63/83), with a complete remission (CR) rate of 44.6% (37/83). In univariate analysis, tumor response after CCRT (p = 0.000), recurrence site (p = 0.028) and concurrent chemotherapy (p = 0.090) showed a trend favoring better OS. Multivariate analysis revealed that tumor response after CCRT (p = 0.000) and concurrent chemotherapy (p = 0.010) were independent predictors of OS. Forty-seven patients had progressive diseases after CCRT, 27 had local failure (27/47, 57.4%), 18 had distant metastasis (18/47, 38.3%) and 2 had both local and distant failures (2/47, 4.3%).

**Conclusions:**

3DCRT-based CCRT is effective in postoperatively recurrent esophageal SCC. Patients that obtained complete remission after CCRT appeared to achieve long-term OS and might benefit from concurrent TP regimen. Local and distant failures remained high and prospective studies are needed to validate these factors.

## Background

Esophageal cancer (EC) remains one of the most fatal malignancies in the world. In 2005, about 497,700 new cases occurred worldwide, and the prevalence is expected to increase by approximately 140% by 2025 [1]. Unlike western countries, in China the predominant histological subtype of EC is squamous cell carcinoma (ESCC), and tumors are more likely to develop in the middle and upper thoracic esophagus [2,3]. Surgical resection is the primary treatment for thoracic ESCC in many cancer institutes as it offers a chance of cure. Although the 5-year overall survival rates of patients who underwent curative tumor resection range from 31 to 55% [4,5], postoperative recurrence remains the major type of failure. The recurrence rate of surgical patients ranges from 36 to 56% and the median time to recurrence ranges from 10 to 12 months; while anastomosis, regional (mediastinum and upper abdomen) lymph node and supraclavicular lymph node are the most common recurrence sites [6-8]. Significant difficulty is often encountered in determining treatment options for recurrent disease after esophagectomy, and patient prognosis is generally poor [9-11].

Although optimal treatment for patients with postoperative recurrence of ESCC has remained controversial, recent advances in anticancer drug and radiation techniques may help to improve treatment outcomes. Since these patients hadn’t received radiotherapy (RT) or chemotherapy before, RT combined with concurrent chemotherapy might have a beneficial symptomatic effect and a possibility to obtain long-term survival [12-16]. Thus, the factors affecting this survival after postoperative recurrence in ESCC patients need to be fully explored. In our study, we evaluated the prognostic factors and treatment failure pattern of concurrent chemoradiotherapy (CCRT) for postoperative recurrence of ESCC.

## Methods

### Acquisition of clinical data

We retrospectively reviewed the records of 83 consecutive patients treated with three- dimensional conformal radiotherapy (3D-CRT)-based CCRT for postoperative recurrence of ESCC between June 2001 and December 2010 in the Sun Yat-Sen University Cancer Center. Patients recruited in our study had: R0 resection (no residual microscopic disease) for primary ESCC with 2-field or 3-field lymphadenectomy; cervical and/or thoracic postoperative recurrence (biopsy proven or 3-month follow-up CT showed subsequent development of disease); no distant organ metastasis or abdominal lymphadenopathy; no history of RT or chemotherapy; ECOG performance ≤3.

Clinical data collected for each patient included age, sex, thoracic surgery history, primary esophageal tumor location, stage and histology of primary ESCC, Eastern Cooperative Oncology Group (ECOG) performance status, interval time between surgery and recurrence, recurrence sites, histology of recurrent lesions, irradiation dose, concurrent chemotherapy regimens and tumor response to CCRT. The 7th edition of American Joint Committee on Cancer (AJCC) staging system for esophageal cancer released in 2010 was used to restage the primary diseases after radical surgery. Written informed consent was obtained from the patient for the publication of this report and all accompanying images.

### Treatment

Our techniques for patient immobilization, simulation and treatment planning were performed according to standard protocol for esophageal carcinoma receiving 3-DCRT in our department [17]. With the patient in supine position, a cradle for immobilization was made with vacuum. Individual patient was scanned from the Atlas (C1) to the second lumbar vertebra (L2) level to cover the whole neck, lung, esophagus and celiac lymph node regions. CT scan was performed with 0.5 cm thickness slices. Briefly, the gross tumor volume (GTV) consisted of recurrent lesion diagnosed by biopsy or subsequent CT scan; the regions of tumor described on endoscopy but not seen on CT were also included in the GTV. To minimize interobserver variability, CT scans of all patients were reviewed by a single radiologist (Dr. PeiQiang Cai). The criteria of lymph node positivity on the CT scan were: short axis size ≥ 10 mm, lymph node with infiltrative margin, or central necrosis [18]. The clinical target volume (CTV) of patients comprised the anastomosis, supraclavicular, and station 1–5 and 7 lymph nodes [19]. Two planning target volume (PTV) had been defined. PTV1 was defined as the GTV plus a 0.5 cm margin and PTV2 was defined as the CTV plus a 0.5 cm margin in all direction, respectively. All patients had 3D-CRT treatment plan calculated by Pinnacle planning system, and treated with a 6-MV linear accelerator. The median dose was 60 Gy to PTV1 (range from 56-68 Gy), and 46 Gy to PTV2 (range from 40 to 54 Gy). Dose constraint for critical organs: spinal cord < 46 Gy, mean lung dose < 17 Gy and V20 <30%.

Forty-two patients were treated with 2 cycles of cisplatin and 5-fluorouracil concurrently with RT. 18 patients received chemotherapy consisted of 60 mg/m^2^ of cisplatin administered on Days 1 and 29, 300 mg/m^2^/24 h of 5-Fu administered on Days 1 to 3 and Days 29 to 31. Twenty-two patients received cisplatin and 5-fluorouracil regimen consisted of 30 mg/m^2^/day cisplatin and 500 mg/m^2^/day 5-Fu administered on Days 1–5 and 29–33. Another 41 patients were treated docetaxel-based regimens, 26 with 2 cycles of docetaxel and cisplatin, the regimen containing 60 mg/m^2^ docetaxel on Days 1 and 29, and 80 mg/m^2^ cisplatin on Day 1 and 29 [17]; 15 with concurrent chemotherapy comprising cisplatin 30 mg/m^2^ and docetaxel 30 mg/m^2^ weekly for 4–6 weeks [20,21]. The chemotherapy regimens were subsequent institutional standards with PF earlier and taxanes later. Supportive therapy was administered by clinical dietitian, and patients had been evaluated by NRS2002 since 2003.

### Follow-up and response assessment

The beginning of the follow-up period was defined as the last date of CCRT treatment. Patients underwent chest CT scan every 3 months, upper digestive tract endoscopy and abdominal ultrasonography every 6 months for 2 years after the CCRT, and then chest CT scan, endoscopy and abdominal ultrasonography every 6 months for another 3 years. Bone scan was administrated when patients were suspected for bone metastasis. Rates and times of treatment response, overall survival, local relapse and distant metastasis were recorded.

Evaluation of the tumor response was performed 1–3 months after CCRT. Tumor response was recorded according to the definition of Response Evaluation Criteria In Solid Tumors (RECIST). Complete response for the recurrent anastomotic tumor was defined upon endoscopic observation as disappearance of the tumor lesion, ulceration, and absence of cancer cells in biopsy specimens. Complete response for lymph nodes was defined according to the RECIST as the complete disappearance of the lymph nodes. However, lymph nodes of <5 mm or residual connective tissue after disappearance of cancer with no evidence of progression after completion of treatment were regarded as noncancerous tissue [22]. Multiple failures comprised both local and distant failures after CCRT.

### Statistical analysis

The study endpoint was overall survival (OS). OS was calculated as the time from the last date of radiotherapy to the date of death from any cause or to the last visit before April 31, 2011, censored at the date of last follow up. Continuous variables such as age, interval time between surgery and CCRT, RT dose were discretized at the sample median and then analyzed as nominal categorical variables. Each variable was assessed first in a univariate analysis and the variables that reached a P value <0.10 were evaluated in a multivariate analysis. Survival curves were plotted using the Kaplan-Meier method. We fitted the proportional hazards model using Cox regression. After testing for variable interactions, a forward stepwise elimination procedure was used to determine the best-fitting model. P values <0.05 were regarded as statistically significant in multivariate analysis. All statistical analyses were performed using SPSS 19.0 software (IBM).

## Results

### Patient characteristics

Patient characteristics are detailed in Table 1. The study included 83 patients and comprised 21 female and 62 male. Seventy-three patients (88.0%) had ECOG performance status of 0–1. The locations of primary esophageal cancer removed by radical surgery varied, with most lesions (62/83, 74.7%) locating at the middle thoracic esophagus. Histology of primary tumor of all the 83 patients was ESCC and 50 (60.2%) had G1-2 diseases. Forty-five patients had biopsies for recurrent lesions with histology showing ESCC. Thirty-eight patients had recurrent disease diagnosed only by follow-up CT but without biopsy, 18 had recurrent diseases of station 1 nodes (10 with supraclavicular lymph nodes and 8 with cervical paraesophageal nodes), 20 had enlarged mediastinal station 2 and 4 nodes. The median interval time between surgery and CCRT was 14 month (range, 2–88 months). Twenty patients (24.1%) had anastomotic recurrence (AR) with or without locoregional lymphadenopathy, 63 (75.9%) had mediastinal and/or supraclavicular lymph node recurrence (LR). The most common involved lymph node stations were 2R (26/83, 31.3%), 4R (19/83, 22.9%), 1R (11/83, 13.3%) and 1 L (14/83, 16.9%). Median dose of RT was 60 Gy (range, 56-68 Gy).

**Table 1:** Characteristics of patients

**Characteristics**	**Patients (n = 83) No. (%)**
Sex	
Male	62 (74.7%)
Female	21 (25.3%)
Age (year), median (range)	55, (37–80)
ECOG performance status	
0-1	73 (88.0%)
2-3	10 (12.0%)
Primary tumor location	
Upper	9 (10.8%)
Middle	62 (74.7%)
Lower	12 (14.5%)
Radical surgery	
Three-field resection	11 (13.3%)
Two-field resection	72 (86.7%)
Histology of primary tumor (SCC)	
G1-2	50 (60.2%)
G3-4	33 (39.8%)
Stage of primary tumor (7th edition)	
IA-IB	8 (9.6%)
IIA-IIB	38 (45.8%)
IIIA-IIIC	37 (44.6%)
Time between surgery and RT(month), median (range)	14 (2–88)
Recurrence site	
Anastomosis with/without lymphadenopathy	20 (24.1%)
Supraclavicular and/or regional lymph node	63 (75.9%)
RT dose (Gy), median (range)	60 (50–68)
Concurrent chemotherapy	
cisplatin + 5-Fu	39 (47.0%)
Docetaxel + cisplatin	44 (53.0%)
Tumor Response after CCRT	
CR	37 (44.6%)
PR	26 (31.3%)
SD	11 (13.3%)
PD	9 (10.8%)

### Treatment outcome

**Figure 1:**
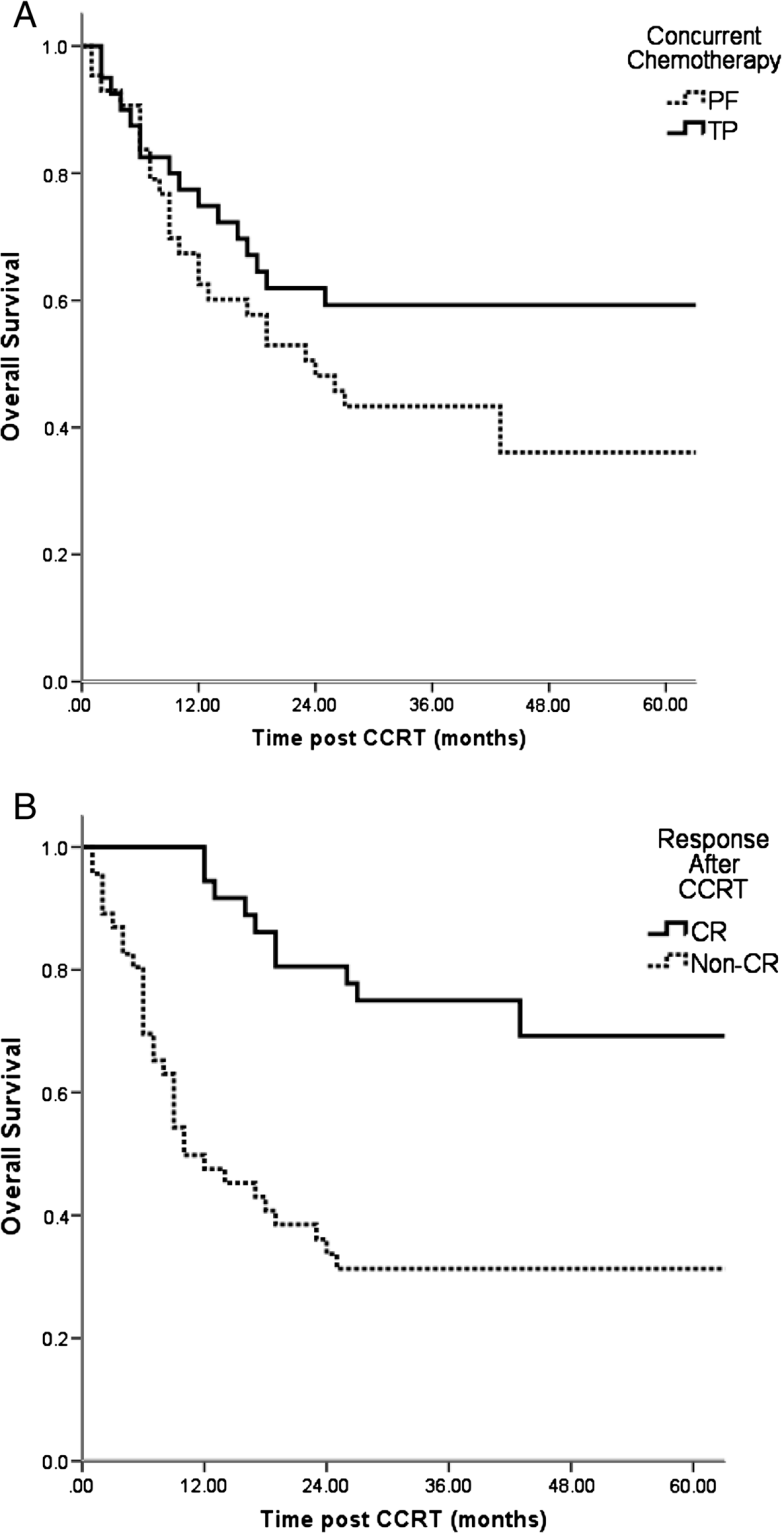
Overall survival of patients with different treatment responses and concurrent chemotherapy regimens (A) OS of patients treated by different concurrent chemotherapy regimens, (B) OS of patients with different treatment responses.

All patients were treated with 3D-CRT and concurrent chemotherapy, 39 received concurrent PF, and 44 received TP. With a median follow-up of 34 months (range, 2–113 months), the 3-year overall survival (OS) of all the patients was 51.8%, median OS time was 43.0 months. Univariate analysis showed that tumor response after CCRT (p = 0.000) and recurrence site (p = 0.028) had significant associations with OS, while concurrent chemotherapy (p = 0.090) showed a trend of association with OS (Table 2). Clinical factors that were statistically significant (p < 0.10) in a univariate analysis were analyzed further in a multivariate analysis with a stepwise selection of variables. Only patients that had tumor response after CCRT (p = 0.000) and concurrent chemotherapy (p = 0.010) were selected by a stepwise selection as factors in the final models (Table 3). The overall tumor response rate was 75.9% (63/83), with a complete remission (CR) rate of 44.6% (37/83) and partial remission (PR) rate of 31.3% (26/83). The 3-year OS of CR and non-CR patients were 75.7% and 35.6%, while it was 59.2% for patients received concurrent TP chemotherapy and 43.3% for concurrent PF, respectively (Figure 1).

**Table 2:** Univariate analysis of prognostic factors of overall survival (n = 83)

**Variable**	**HR, 95% CI**	***p *** **value**
Sex (male vs. female)	0.70 (0.26-1.89)	0.477
Age (>55 yrs vs. ≤55 yrs)	0.67 (0.28-1.57)	0.344
ECOG performance status (0–1 vs. 2–3)	0.72 (0.27-1.86)	0.523
Primary tumor location (Upper vs. Middle vs. Lower)	0.80 (0.40-1.61)	0.533
Radical surgery (Two-field vs. three-field)	1.31 (0.55-3.12)	0.540
Histology of primary tumor (G1-2 vs. G3-4)	1.05 (0.51-2.13)	0.905
Stage of primary tumor (I-II stage vs. III stage)	1.66 (0.88-3.12)	0.120
Time between surgery and RT (>14 mon vs. ≤14 mon)	0.77 (0.34-1.77)	0.538
Recurrence site (AR vs. LR)	2.70 (1.06-6.88)	0.028^*^
RT dose(>60 Gy vs. ≤60 Gy)	1.26 (0.47-3.35)	0.648
Concurrent chemotherapy (PF vs. TP)	0.58 (0.31-1.09)	***0.090***^***#***^
Tumor response after CCRT (CR vs. non-CR)	4.20 (2.04-8.65)	0.000^*^

Note: *Univariate analysis showed that tumor response after CCRTand recurrence site had significant associations with OS. # Univariate analysis showed that concurrent chemotherapy showed a trend of association with OS.

**Table 3:** Multivariate analysis of prognostic factors for overall survival (n = 83)

**Variable**	**HR, 95% CI**	***p *** **value**
Tumor response after CCRT (CR vs. non-CR)	5.10 (2.33-11.15)	0.000^*^
Concurrent chemotherapy (PF vs. TP)	0.39 (0.19-0.80)	0.010^*^

Note: *Multivariate analysis showed that tumor response after CCRT and recurrence site had significant associations with OS.

### Failure pattern

Forty-one patients died during the observation period of this study, 47 had progressive diseases after CCRT, 27 had local failure (27/47, 57.4%), 18 had distant metastasis (18/47, 38.3%) and 2 had both local and distant failures (2/47, 4.3%). The cause of death was related to progression of disease in 36 patients and to unknown reasons in 5 patients. Table 4 demonstrated the failure pattern of CCRT. Lung (11/20) was the most common site of metastasis, while other sites consisted in: bone (8/20), liver (5/20), brain (2/20), and axillary node (1/20).

**Table 4:** Relationship between tumor response, concurrent chemotherapy and patterns of treatment failure (n = 83)

**Characteristic**	**Persistent local disease (%)**	**Locoregional recurrence (%)**	**Distant metastasis (%)**	**Multiple failures (%)**
Tumor response				
CR (n = 37)	0 (0%)	6 (16.2%)	5 (13.5%)	1 (2.7%)
Non-CR (n = 46)	9 (19.6%)	12 (26.1%)	13 (28.3%)	1 (2.2%)
Concurrent chemotherapy				
PF (n = 39)	6 (15.4%)	12 (30.8%)	9 (23.1%)	1 (2.6%)
TP (n = 44)	3 (6.8%)	6 (13.6%)	9 (20.5%)	1 (2.3%)
Total (n = 83)	9 (10.8%)	18 (21.7%)	18 (21.7%)	2 (2.4%)

### Toxicities

The most frequent toxicities observed were vomiting, neutropenia, esophagitis, cough, and the large majority of toxicity degrees were Grade (G) 1 or 2. G3 vomiting was observed in 8 out of 39 patients (30.8%) who received concurrent cisplatin plus 5-Fu, and in 10 out of 44 patients (33.3%) who received docetaxel plus cisplatin regimen. G3 neutropenia was more common in docetaxel-based group, where occurred in 11 out of 44 patients (36.7%) compared with 6 out of 39 patients (23.1%) in cisplatin plus 5-Fu group. G3 esophagitis had been observed in 2 patients (2/39, 5.1%) in cisplatin plus 5-Fu group and 3 out of 44 patients (6.8%) in docetaxel-based group. These 5 patients had a nasogastric feeding tube inserted due to pain or difficulty in swallowing. No G3 or higher cough was recorded among all the patients. There were no treatment-related deaths and pneumonitis.

## Discussion

CCRT has been established as a curative alternative for the treatment of ESCC, conferring substantial improvement in survival compared with RT alone. However, the optimal treatment of recurrent ESCC after surgery remained controversial. Some studies on the effectiveness of radiotherapy with or without chemotherapy for treatment of postoperative recurrent ESCC reported 2-year survival rates of only 15-31% with short-term observation. Lu et al. reported that the OS of all 73 recurrent ESCC patients was 46.7% and 4.7% at 1 and 3 years respectively, and patients receiving CCRT had better OS than those receiving RT alone with 1-year OS 62.5% in CCRT group vs. 33.8% in RT alone group, 3-year OS 10.5% in CCRT group vs. 0% in RT alone group [12]. A recent phase II study on radiotherapy combined with nedaplatin and 5-Fu for postoperative loco-regional recurrent ESCC showed improved 5-year OS of 27.0%. High objective response rate (76.7%) for recurrent tumors was obtained in the study, suggesting a potential benefit of concurrent chemotherapy [23].

In our study, the ESCC patients with localized recurrence treated by CCRT showed a promising improvement of prognosis compared to the previous reports in which the 3-year OS rate of all the patients was 51.8%, the median OS time was 43.0 months, and the overall tumor response rate was 75.9% (63/83). Similar to primary localized ESCC, prolonged survival is unlikely to be achieved in patients who had postoperative recurrent ESCC unless they have obtained a good local response to initial chemoradiotherapy. In our study, an improvement in survival was observed in patients who obtained CR after CCRT, with the 3-year OS of CR and non-CR patients being 75.7% and 35.6% (p = 0.000). Recent studies of preoperative CCRT followed by surgery showed that the excellent prognosis were associated with the patients who achieve a complete pathological response to preoperative CCRT [24-26]. Although clinical CR after CCRT may not be significantly correlated to pathologic CR [27], maximizing the CR rate is likely to increase the proportion of patients with most favorable outcome and potentially affect survival of the group as a whole. In our study, it showed that in the CR group (37 patients), 7 patients developed in-field local recurrence and 6 patients had distant metastases; while in the non-CR group (46 patients) 22 had local recurrence and 14 had distant metastases. These results suggested that the survival benefit in CR group mainly came from the decrease of the rate of local recurrence. Moreover, local recurrence might have a relationship with the occurrence of distant metastasis.

Our results also demonstrated a benefit in survival for patients treated with a regimen of docetaxel-based concurrent chemotherapy. The 3 year OS rate was 59.2% for patients received concurrent TP chemotherapy and 43.3% for concurrent PF. The Swiss Group for Clinical Cancer Research (SAKK) established a preoperative induction schedule comprising docetaxel and cisplatin followed by CCRT [28]. This schedule proved to be effective and feasible, resulting in a 3-year survival rate of 53%. Indeed, taxanes promote tubulin conjugation and stabilize microtubule formation, thereby inhibiting mitosis. In addition to their cytotoxic effect, taxanes also act as excellent radiosensitizers, arresting the cell cycle in the G2/M phase [29]. Multiple studies have examined the use of docetaxel and platinum compounds as radiosensitizers for esophageal cancer and neoadjuvant chemotherapy, reporting high pathological CR rates [30,31]. Zanoni et al. reported that the results of neoadjuvant concurrent radiotherapy with docetaxel, cisplatin, and 5-Fu was a promising regimen, with a 41.9% pathological CR rate and a good safety profile [32]. Encouraged by few recent studies suggesting the efficacy of taxanes in esophageal cancer, investigators performed a phase II trial to evaluate the feasibility and safety of docetaxel and cisplatin in 5-FU and cisplatin-pretreated esophageal cancer. A total of 38 patients were enrolled, and 35 patients were available for evaluation. The median and total numbers of cycles delivered were 3.5 (range, 1–9 cycles) and 162, respectively. One patient (2.6%) achieved complete response, 12 (31.6%) achieved partial response, 12 (31.6%) had stable disease, and 10 (26.3%) had progressive disease. The overall response rate was 34.2% (95% confidence interval, 19.6-51.3). The median progression-free survival and overall survival times were 4.5 ± 1.3 months (95% CI, 4.1-4.9) and 7.4 ± 0.4 months (95% CI, 7.3-7.5), respectively. The best results were observed in patients who had good performance status at baseline and who had longer treatment-free intervals after first-line chemotherapy [33]. In our study, 10 out of 83 patients (12.0%) had ECOG 2–3. Among them 6 patients received 2 cycles of cisplatin and 5-fluorouracil concurrently with RT, and 4 patients received concomitant weekly docetaxel and cisplatin for 4–6 weeks. This suggested that performance status at baseline is an important issue in choosing a concurrent chemotherapy regimen.

Another interesting finding in our study was that AR patients had a better OS compared with LR patients (3-year rate 74.4% vs. 43.6%, P = 0.028). Among the 20 AR patients, 11 patients had recurrent lesion only at anastomosis, 7 patients had anastomotic recurrence and one station lymph node involvement, and 2 patients had anastomotic recurrence and more than one stations lymph node involvement. After CCRT, 1 patient developed in-field local recurrence and 5 had distant metastases in the 20 AR patients; while 26 had local recurrence and 15 had distant metastases among the 63 LR patients. The recurrent lesion in AR patients seemed to be more localized and had less lymph node involvements. This might because the anastomotic recurrence is more likely to cause dysphagia and be diagnosed earlier. Other than that, anastomotic recurrence could be regarded as a second primary cancer; the prognosis would be different from extensive lymph node involvement.

The higher treatment response and local control rates in this study compared with other published data might be due to: 1) the different treatment target definition (the CTV comprised the anastomosis, supraclavicular, and station 1–5 and 7 lymph nodes in order to involve all the subclinical metastatic regions); 2) the use of 3D-CRT technique (3D-CRT had better GTV high dose coverage than two-dimensional conventional radiotherapy (2DCRT) which had been used by most previous studies); 3) the higher irradiation dose (median dose in present study was 60 Gy, range from 56 to 68 Gy. Zhang’s study suggested that higher dose of 60 Gy could achieve better local control) [34]. 4) 44 out of 83 (53.0%) patients receive docetaxel-based concurrent chemotherapy. This retrospective study has several limitations such as selection bias, various chemotherapy regimens, small study numbers and short follow-up time. Moreover, instrumental diagnostic procedures such as endoscopic ultrasound (EUS) and positron emission tomography (PET) were not used for a precise CR evaluation of the recurrent lesions after CCRT. However, our results revealed that ESCC patients with postoperative localized recurrent disease could achieve promising improvement in outcome when treated with CCRT and they have the potential to be cured. Patients could benefit from proper target definition, 3D-CRT technique and aggressive concurrent chemotherapy.

## Conclusions

3D-CRT-based CCRT is effective and well-tolerated in patients with recurrent ESCC; patients obtaining complete remission after CCRT or receiving concurrent TP chemotherapy appeared to achieve long-term OS. Although local and distant failures remained high in the whole group, patients with localized disease could still benefit from aggressive CCRT. Prospective studies are needed to validate these predictive factors.

## Abbreviations

3DCRT: Three-dimensional conformal radiotherapy; CCRT: Concurrent chemoradiotherapy; SCC: Squamous cell carcinoma; PF: Cisplatin plus 5-fluorouracil; TP: Docetaxel plus cisplatin; OS: Overall survival; CR: Complete remission; RT: Radiotherapy; ECOG: Eastern Cooperative Oncology Group; AJCC: American Joint Committee on Cancer; GTV: Gross tumor volume; CTV: Clinical target volume; PTV: Planning target volume; RECIST: Response evaluation criteria in solid tumors; PR: Partial remission; AR: Anastomotic recurrence; LR: Lymph node recurrence; 2DCRT: Two-dimensional conventional radiotherapy; EUS: Endoscopic ultrasound; PET: Positron emission tomography.

## Competing interests

The authors declare that they have no competing interests.

## Authors’ contributions

YB carried out data analysis and drafted the manuscript. SLL collected clinical data and drafted the manuscript. QCZ collected clinical data and data analysis. PQC reviewed all CT and PET-CT images. SA carried out data analysis and English editing. QQL participated patients treatment and clinical care. HYH participated patients treatment and clinical care. MZL participated patients treatment and clinical care. JHF participated patients treatment and clinical care. THR participated patients treatment and clinical care. QLparticipated patients treatment and clinical care. HL carried out data analysis, drafted the manuscript and participated patients treatment and clinical care. All authors read and approved the final manuscript.
